# Gender-affirming care, mental health, and economic stability in the time of COVID-19: A multi-national, cross-sectional study of transgender and nonbinary people

**DOI:** 10.1371/journal.pone.0254215

**Published:** 2021-07-09

**Authors:** Brooke A. Jarrett, Sarah M. Peitzmeier, Arjee Restar, Tyler Adamson, Sean Howell, Stefan Baral, S. Wilson Beckham

**Affiliations:** 1 Department of Epidemiology, Johns Hopkins School of Public Health, Baltimore, MD, United States of America; 2 Department of Health Behavior and Biological Sciences, University of Michigan School of Nursing, Ann Arbor, Michigan, United States of America; 3 Department of Health, Policy, and Management, Johns Hopkins School of Public Health, Baltimore, MD, United States of America; 4 Hornet, San Francisco, CA, United States of America; 5 Department of Health, Behavior, and Society, Department of International Health, Johns Hopkins School of Public Health, Baltimore, MD, United States of America; University of California San Diego, UNITED STATES

## Abstract

**Background:**

Transgender and nonbinary people are disproportionately affected by structural barriers to quality healthcare, mental health challenges, and economic hardship. This study examined the impact of the novel coronavirus disease (COVID-19) crisis and subsequent control measures on gender-affirming care, mental health, and economic stability among transgender and nonbinary people in multiple countries.

**Methods:**

We collected multi-national, cross-sectional data from 964 transgender and nonbinary adult users of the Hornet and Her apps from April to August 2020 to characterize changes in gender-affirming care, mental health, and economic stability as a result of COVID-19. We conducted Poisson regression models to assess if access to gender-affirming care and ability to live according to one’s gender were related to depressive symptoms, anxiety, and changes in suicidal ideation.

**Results:**

Individuals resided in 76 countries, including Turkey (27.4%, n = 264) and Thailand (20.6%, n = 205). A majority were nonbinary (66.8%, n = 644) or transfeminine (29.4%, n = 283). Due to COVID-19, 55.0% (n = 320/582) reported reduced access to gender-affirming resources, and 38.0% (n = 327/860) reported reduced time lived according to their gender. About half screened positive for depression (50.4%,442/877) and anxiety (45.8%, n = 392/856). One in six (17.0%, n = 112/659) expected losses of health insurance, and 77.0% (n = 724/940) expected income reductions. The prevalence of depressive symptoms, anxiety, and increased suicidal ideation were 1.63 (95% CI: 1.36–1.97), 1.61 (95% CI: 1.31–1.97), and 1.74 (95% CI: 1.07–2.82) times higher for individuals whose access to gender-affirming resources was reduced versus not.

**Discussion:**

The COVID-19 crisis is associated with reduced access to gender-affirming resources and the ability of transgender and nonbinary people to live according to their gender worldwide. These reductions may drive the increased depressive symptoms, anxiety, and suicidal ideation reported in this sample. To improve health of transgender and nonbinary communities, increased access to gender-affirming resources should be prioritized through policies (e.g., digital prescriptions), flexible interventions (e.g., telehealth), and support for existing transgender health initiatives.

## Introduction

The pandemic caused by the SARS-CoV-2 virus has resulted in more than 167 million cases of novel coronavirus disease (COVID-19) and over three million deaths [1]. In response, countries have implemented a wide range of measures to quell transmission— shelter-in-place orders, closures of business and schools, and the cancellation of surgeries perceived to be elective [2]. While these interventions are focused on reducing COVID-19 cases and increasing healthcare capacity, they have also negatively affected healthcare access, mental health, and economic stability worldwide [3–6].

A growing body of literature describes global interruptions to prescriptions for diseases like HIV, increases in depression, and significant losses of job, insurance, and food security as a result of COVID-19 [7–11]. However, recent research has demonstrated that these effects disproportionately burden groups that are already marginalized. Specifically, COVID-19 control measures have been associated with heightening existing health disparities and social inequities along lines of poverty and occupation, race and ethnicity, and sexual orientation [12–16]. The COVID-19 crisis may also uniquely affect transgender and nonbinary people.

Prior to the emergence of COVID-19, transgender and nonbinary individuals experienced barriers to care, greater mental health challenges, and economic vulnerabilities caused by stigma, discrimination, and minority stress [17–19]. Transgender and nonbinary populations face a scarcity of clinicians trained in gender-affirming practices and widespread transphobia among healthcare staff, both of which make healthcare less accessible [20–22]. Yet access to gender-affirming healthcare (e.g., chest/breast surgery), services (e.g., hair removal), and goods (e.g., binders and packers) can substantially improve the quality of life and mental health of transgender and nonbinary populations, who frequently have elevated levels of depression, anxiety, and suicidal ideation [23–26]. A disproportionate number of transgender and nonbinary individuals also experience structural vulnerabilities, such as economic, food, and housing insecurity, that can reinforce or worsen barriers to gender-affirming resources and mental health counseling [27, 28].

While the COVID-19 crisis has been associated with negative impacts to the lives of many, its effects are likely further exacerbating the aforementioned existing risks among transgender and nonbinary individuals. The COVID-19 crisis has also had unique impacts on the transgender community. For example, there have been documented cancellations and delays in gender-affirming surgeries [29]; such delays and cancellations have previously been connected to negative mental health consequences [23, 30]. Furthermore, many transgender and nonbinary individuals who were living according to their gender prior to the emergence of COVID-19 have had to return to living according to their sex assigned at birth upon moving in with relatives [31]. Researchers have also reported on gendered policies from Panama, Peru, and Columbia that attempted to reduce the density of crowds in public places by requiring women and men to access essential services on alternating days— a policy that, like other gender-based laws, would likely result in violence against transgender communities [32]. In these situations, transgender and nonbinary individuals are limited in their ability to live safely and comfortably as themselves. The impact COVID-19 is likely to be especially adverse for those who are already economically marginalized, occupying other marginalized identities (e.g., people who are racial or ethnic minorities, living with HIV, or living with disabilities), or both.

Of the studies to date about the impact of COVID-19 on transgender and nonbinary individuals, a majority have been conducted in a single country like the United States and focused on measuring a narrow spectrum of indicators. The objective of this study was to describe the impact of the COVID-19 crisis on gender-affirming care, mental health, and economic stability among transgender and nonbinary individuals in countries across the world. We also examined the association between reduced access to gender-affirming care and ability to live according to one’s gender with multiple mental health indicators.

## Methods

### Study design and participants

For this cross-sectional study, we used data from a survey from the COVID Disparities Working Group. The survey was distributed between April 16 and August 3, 2020 via Hornet and Her— social networking apps marketed to sexual minorities, both cisgender and transgender, with more than 4 million monthly active users across 196 countries in six continents. We sent survey invitations to the app-specific inboxes of any user who had signed into the app in the past year. Individuals voluntarily opted to take the survey. To begin the survey, individuals had to report being 18 years or older, view a consent form, and indicate their informed consent by clicking a button to begin. The Johns Hopkins School of Public Health Institutional Review Board reviewed the survey study, approving it with a Category 4 exemption.

A total of 24,618 individuals began the survey. For these analyses, we included 1,285 transgender and nonbinary adults, which we defined as people 18 years or older who self-reported being transgender, nonbinary, or a gender different than their sex assigned at birth. We excluded all women assigned female or intersex at birth (n = 161), all men assigned male or intersex at birth (n = 12,740), individuals who did not report a gender (n = 9,751), individuals who only reported not knowing, not wishing to disclose, or being unable to disclose their gender (n = 654), and non-transgender identifying men and women who did not report an assigned sex at birth (n = 27).

To ensure the quality of our study population, we screened for duplicate survey responses based on IP address, and again by searching for identical responses to twenty randomly selected variables but found none. We also excluded individuals who completed 89% or less of the survey (n = 271), who finished in less than the minimum piloted time of seven minutes (n = 47), or provided conflicting responses for multiple questions (n = 3) for a final sample size of 964. Relevant data are available in the Zenodo repository [33].

### Demographic measures

Individuals self-reported gender by choosing any number of the following options: woman, man, transgender man, transgender woman, or nonbinary (including gender-diverse, genderqueer, gender nonconforming, gender expansive, and agender). They also self-reported their country of residence, age, socioeconomic status, years of education, ethnic minority and immigration status, access to masks during the COVID-19 pandemic, and whether the government in their area had ever imposed confinement orders (e.g., mobility restrictions to stay-at-home). We categorized countries according to regions defined by the World Health Organization.

We used eight mutually exclusive categories to describe reported genders. To increase the power for our analyses, we then collapsed individuals into three groups, building on recommendations from Reisner et al: (1) transmasculine, i.e., people who were assigned female at birth (AFAB) or intersex who self-reported being transgender or being a man; (2) transfeminine, i.e., people who were assigned male at birth (AMAB) or intersex who self-reported being transgender or being a woman; and (3) nonbinary, i.e., individuals who reported being either solely nonbinary, both a man and a woman, or a transgender man and transgender woman.

We chose to operationalize three gender categories for multiple reasons. First, while some nonbinary individuals explicitly reported also being transgender, the majority did not. We wanted to honor this distinction while also allowing for individuals who reported being both men and women to transcend the transfeminine versus transmasculine binary. Second, our survey presented a limited number of gender options to an audience from multiple countries in which being a third gender (e.g., two-spirit, bissu, fa’afafine) is distinct from many Western concepts of being transgender [34–37]. Lastly, in line with recommendations from Restar et al., we saw statistically significant differences when comparing nonbinary individuals with transmasculine and transfeminine individuals [38]. Therefore, presenting results stratified by gender (i.e., a “gender-specific” approach) was more appropriate than conducting analyses on all individuals together and presenting them as a single population (i.e., a “gender-inclusive” approach).

### COVID-19 impact measures

Individuals answered questions about the impact of COVID-19 and the resulting response on their access to gender-affirming resources, their mental health, and their economic stability. For indicators related to gender-affirming resources, we asked those who self-identified as transgender or nonbinary (n = 865) whether the COVID-19 crisis limited their access to the following: hormone therapy and/or medications; surgical aftercare materials (e.g., vaginal dilators); cosmetic supplies and services (e.g. makeup, wigs, and hair removal); mental health counseling and therapy services; and body modifiers (e.g., binders and packing materials); to which they could respond, “Yes,” “No,” or “Not Applicable.” We characterized the severity of interruptions to gender-affirming care by whether individuals reported that more than one resource had been impacted. We also asked whether the COVID-19 crisis had changed the amount of time that the individual could live according to their gender (“Compared to before the COVID-19 crisis, how often are you able to live according to your gender identity?”). We categorized the five-point Likert scale into three categories: More than before (i.e., more or a lot more as compared to before COVID-19), about the same as before COVID-19, and less than before (i.e., less than or not at all as compared to before COVID-19).

For mental health indicators, we used the 4-item patient health questionnaire (PHQ-4) to screen for common symptoms of depression and anxiety, which we dichotomized with a score of three or more being considered positive [39]. We assessed the impact of COVID-19 on loneliness (“Have you been feeling lonely since the COVID crisis began?”) using a four-point Likert Scale, which we dichotomized into a positive sentiment (“not lonely” or “not much lonely”) and negative sentiment (“very much lonely” or “a little lonely”). We also asked how often they had thought about taking their own life presently and in the six months prior to the COVID-19 pandemic with the following answer options: “never,” “seldom,” “quite often,” “very often,” and “all the time.” We created four categories to describe changes from pre- to mid-pandemic: was and remains rare (i.e., “never” or “seldom”), was and remains frequent (i.e., “quite often,” “very often,” or “all the time”), decreased, or increased. To characterize resiliency, we asked about the following in the face of the COVID-19 crisis: if they had “sources of hope, strength, comfort, and peace”; if they were “intent on finding emotional support and therapy for themselves”; and if they believed that they were able to “live a happy, full life despite the crisis.”

### Statistical analyses

We conducted descriptive analyses of demographic measures and presented descriptions of the COVID-19 impact measures stratified by gender, using chi-squared and Fisher’s exact tests as appropriate. We also stratified results by country in the supplementary materials. We used Poisson regression models with complete case analyses to assess for differences in the prevalence of screening positive for depression and anxiety among individuals with reduced (versus continued) access to gender-affirming resources and individuals who reported being able to live according to their gender less (versus more) since the COVID-19 pandemic started. We reported these comparisons as prevalence ratios. We used the same approach to assess the impact of reductions in access to care and changes in ability to live according to one’s gender on changes in suicidal ideation, stratified on baseline levels of suicidal ideation. We conducted all analyses in Stata version 14 [40].

## Results

These analyses primarily consisted of nonbinary (66.8%, n = 644/964) and transfeminine (29.4%, n = 283) individuals (**Table 1**). About 47% (n = 451) were from the European region and 25.1% (n = 242) were from the South-East Asia region. There were 76 countries represented in the sample; a majority of individuals were residents of Turkey (27.4%, n = 264), Thailand (20.6%, n = 199), and Russia (11.5%, n = 117). No other single country accounted for more than 5% of the sample. Individuals were young and highly educated, with 50.5% (n = 487) being between 18 and 29 years old and 42.6% (n = 410) having at least a university degree. Few (12.7%, n = 122) had ever lacked access to a mask during the COVID-19 pandemic and 75.6% (n = 729) lived in a country that had issued COVID-related confinement or “stay-at-home” orders.

**Table 1:** Demographics for transgender and nonbinary individuals from the COVID disparities working group survey distributed by the Hornet and Her apps between April 16 and August 3, 2020 (N = 964).

	Overall (%)^a^
**Self-Reported Transgender Identity**	
Transgender man	95 (9.9%)
Transgender woman	201 (20.9%)
Nonbinary (NB) only	594 (61.6%)
NB transgender woman	17 (1.8%)
NB transgender man	7 (0.7%)
NB, transgender woman, and transgender man	2 (0.2%)
Transgender man and transgender woman	7 (0.7%)
Man and woman	41 (4.3%)
**Researcher-Generated Gender Categories**	
Transmasculine (assigned female sex or intersex at birth)	37 (3.8%)
Transfeminine (assigned male sex or intersex at birth)	283 (29.4%)
Nonbinary^b^	644 (66.8%)
**World Health Organization Region**	
Europe	451 (46.8%)
South-East Asia	242 (25.1%)
Americas	86 (8.9%)
Eastern Mediterranean	85 (8.8%)
Western Pacific	40 (4.2%)
Africa	35 (3.6%)
**Age**	
18–29 years	487 (50.5%)
30–39 years	287 (29.8%)
40–49 years	132 (13.7%)
50+ years	58 (6.0%)
**Socioeconomic Status**	
Lower	151 (15.7%)
Lower Middle	472 (49.0%)
Upper middle	290 (30.1%)
Upper	46 (4.8%)
**Years of Education**	
Less than 6 years	54 (5.6%)
6–12 years	176 (18.3%)
Some university, no degree	193 (20.0%)
Trade school	120 (12.5%)
University degree or more	410 (42.6%)
**Ethnic Minority**	
Yes	250 (26.0%)
No	493 (51.2%)
Don’t know / can’t answer	211 (21.9%)
**Immigrant**	
Yes	143 (14.8%)
No	719 (74.6%)
Not sure	85 (8.8%)
**COVID-19 Crisis Environment**	
Ever lacked access to a mask	122 (12.7%)
In a location that ever issued "stay-at-home" confinement orders	729 (75.6%)

^a^ Denominators include those for whom data were missing: 25 did not report a country, 5 did not report a socioeconomic status, 11 did not report an education level, 10 did not report if they were an ethnic minority, 17 did not report if they were an immigrant, 6 did not report their access to masks, and 7 did not report whether their country of residence had implemented "stay-at-home" confinement orders

^b^ Of those whose gender was nonbinary, 28 reported being assigned female sex at birth, 578 reported being assigned male sex at birth, 37 reported being assigned intersex at birth, and one did not report their sex assigned at birth.

More than half (55%, n = 320/582) of the sample reported that the COVID-19 pandemic had limited their access to one or more gender-affirming resource (**Table 2**). Mental health counseling and therapy was the most commonly cited resource to be affected (42.9%, n = 192/448), with a somewhat greater proportion of transmasculine individuals reporting reduced access to counseling (61.9%, n = 13/21) than nonbinary (43.0%, n = 122/284) and transfeminine (39.9%, n = 57/143) individuals (p-value = 0.16). Transmasculine and transfeminine individuals were more likely than nonbinary individuals to report that the COVID-19 pandemic limited their access to gender-affirming hormones and medications (55.0% [n = 11/20] and 42.1% [n = 61/145] vs. 30.1% [n = 71/236], p-value = 0.01) as well as surgical aftercare materials (42.9% [n = 6/14] and 40.2% [n = 51/127] vs. 28.8% [n = 62/215], p-value = 0.08). All geographic regions reported reductions in access to gender-affirming resources; at least half of the individuals in each region, beside the Western Pacific, reported reduced access to one or more resource (**S1 Table**). More than a third (38.0%, n = 327/860) of individuals reported that the COVID-19 pandemic had reduced or completely eliminated their ability to live according to their gender, with more transfeminine individuals (43.1%, n = 100/232) being unable to living according to their gender as compared to transmasculine (28.6%, n = 8/28) and nonbinary (36.5%, n = 219/600) individuals (p-value <0.001).

**Table 2:** Access to and actualization of gender-affirming resources among self-identified transgender and nonbinary individuals from the COVID disparities working group survey between April 16 and August 3, 2020 (N = 964).

	Overall (%)	Transmasculine	Transfeminine	Nonbinary	p-value^b^
**Experienced reduced access to one or more gender-affirming resource below**^**a**^	320 / 582 (55.0%)	17 / 26 (65.4%)	110 / 189 (58.2%)	193 / 367 (52.6%)	0.25
Hormone therapy and/or gender-affirming medication	143 / 401 (35.7%)	11 / 20 (55.0%)	61 / 145 (42.1%)	71 / 236 (30.1%)	0.01
Surgical aftercare	119 / 356 (33.4%)	6 / 14 (42.9%)	51 / 127 (40.2%)	62 / 215 (28.8%)	0.08
Cosmetic supplies and services, e.g., makeup, wigs, and hair removal	189 / 500 (37.8%)	9 / 21 (42.9%)	65 / 162 (40.1%)	115 / 317 (36.3%)	0.63
Mental health counseling and therapy*	192 / 448 (42.9%)	13 / 21 (61.9%)	57 / 143 (39.9%)	122 / 284 (43.0%)	0.16
Body modifiers, e.g., binders and packing material	160 / 443 (36.1%)	8 / 18 (44.4%)	57 / 148 (38.5%)	95 / 277 (34.3%)	0.52
**Compared to before the COVID-19 pandemic, how often able to live according to their gender**					
More or a lot more	67 / 860 (7.8%)	7 / 28 (25.0%)	22 / 232 (9.5%)	38 / 600 (7.1%)	< 0.001
About the same	466 (54.2%)	13 (46.4%)	110 (47.4%)	343 (57.2%)	
Less or not at all	327 (38.0%)	8 (28.6%)	100 (43.1%)	219 (36.5%)	

^a^ Denominators excluded participants who were not presented with these questions, did not respond, or said that the resource was not applicable to them.

^b^ p-values were calculated using chi-squared and Fischer’s exact tests as appropriate

About half of the sample screened positive for depression (50.4%, n = 442/877) and anxiety (45.8%, n = 392/856), with a larger proportion of transfeminine individuals reporting these outcomes than transmasculine and nonbinary individuals (**Table 3**). Overall, 10.0% (n = 93/928) reported that suicidal ideation had increased during the COVID-19 pandemic, and 12.5% (n = 116) reported that it had decreased. Transfeminine individuals were more likely to report increases in suicidal ideation (11.6%, n = 31/263) while being less likely to agree with statements of resiliency, such as having sources of hope, strength, comfort, and peace (47.9%, n = 100/209) when contrasted with transmasculine (8.3%, n = 3/36; 71.0%, n = 22/31) and nonbinary (9.5%, n = 59/624; 69.5%, n = 367/528) individuals (p-value = 0.98; p-value < 0.001). Sixteen percent (n = 136/851) of individuals reported that they were not intent on finding emotional support and therapy for themselves during the COVID-19 pandemic. Seventy-seven percent (n = 724/940) of the sample expected a reduction in their income, 17% (n = 112/659) expected to lose health insurance, and 53.4% (n = 428/801) reported not having received financial aid, despite need (**Table 4**). Though 40% (n = 361/900) of individuals overall reported cutting or reducing their meals, fewer nonbinary individuals had done so (34.9% [n = 212/607] versus 51.4% [n = 18/35] of transmasculine individuals and 50.8% [n = 131/258] of transfeminine individuals).

**Table 3:** Mental health and resiliency indicators among transgender and nonbinary individuals from the COVID disparities working group survey between April 16 and August 3, 2020 (N = 964)^a^.

	Overall (%)	Transmasculine	Transfeminine	Nonbinary	p-value^b^
**Screened positive (PHQ-4 ≥3)**					
Depression	442 / 877 (50.4%)	17 / 35 (48.6%)	144 / 245 (58.8%)	281 / 597 (47.1%)	0.01
Anxiety	392 / 856 (45.8%)	17 / 33 (51.5%)	125 / 237 (52.7%)	250 / 586 (42.7%)	0.03
**Felt lonely since COVID-19 began**					
Yes	685 / 957 (71.6%)	28 / 37 (75.7%)	216 / 281 (76.9%)	441 / 639 (69.0%)	0.04
**Frequency of suicidal ideation since COVID-19 vs. 6 months prior**					
Was and remains rare	648 / 928 (69.8%)	26 / 36 (72.2%)	183 / 268 (68.3%)	439 / 624 (70.4%)	0.98
Decreased from frequent to rare	116 (12.5%)	4 (11.1%)	33 (12.3%)	79 (12.7%)	
Increased from rare to frequent	93 (10.0%)	3 (8.3%)	31 (11.6%)	59 (9.5%)	
Was and remains frequent	71 (7.6%)	3 (8.3%)	21 (7.8%)	47 (7.5%)	
**Reported having sources of hope, strength, comfort, and peace**					
Yes	489 / 768 (63.7%)	22 / 31 (71.0%)	100 / 209 (47.9%)	367 / 528 (69.5%)	< 0.001
**Reported being intent on finding emotional support and therapy**^**c**^					
Agree	467 / 851 (54.9%)	20 / 35 (57.1%)	129 / 237 (54.4%)	318 / 579 (54.9%)	0.85
Disagree	136 (16.0%)	5 (14.3%)	43 (18.1%)	88 (15.2%)	
**Reported believing they could live a happy, full life despite the pandemic**^**c**^					
Agree	516 / 847 (60.9%)	26 / 34 (76.5%)	137 / 231 (59.3%)	353 / 582 (60.6%)	0.22
Disagree	120 (14.2%)	2 (5.9%)	40 (17.3%)	78 (13.4%)	

^a^ Denominators excluded individuals who did not respond or reported not knowing their answer unless otherwise noted.

^b^ p-values were calculated using chi-squared and Fischer’s exact tests as appropriate.

^c^ Denominator includes those who stated "neither agree nor disagree"

**Table 4:** Economic indicators among transgender and nonbinary individuals from the COVID disparities working group survey between April 16 and August 3, 2020 (N = 964)^a^.

	Overall (%)	Transmasculine	Transfeminine	Nonbinary	p-value^b^
**Lost job due to COVID-19**	151 / 953 (15.8%)	5 / 37 (13.5%)	53 / 278 (19.1%)	93 / 638 (14.6%)	0.21
**Expected reduction in income**					
0%	216 / 940 (23.0%)	13 / 37 (35.1%)	57 / 270 (21.1%)	10 / 633 (23.1%)	0.33
1–39%	265 (28.2%)	11 (29.7%)	70 (25.9%)	184 (29.1%)	
40–99%	337 (35.8%)	9 (24.3%)	101 (37.4%)	227 (35.9%)	
100%	122 (13.0%)	4 (10.8%)	42 (15.6%)	76 (12.0%)	
**Expected to lose health insurance**^**c**^					
Yes	112 / 659 (17.0%)	7 / 23 (30.4%)	40 / 179 (22.4%)	65 / 457 (14.2%)	0.05
No	408 (61.9%)	11 (47.8%)	104 (58.1%)	293 (64.1%)	
**Financial Aid**					
Received and not needed	22 / 801 (2.8%)	3 / 30 (10.0%)	8 / 229 (3.5%)	11 / 542 (2.0%)	0.08
Not received but not needed	159 (19.8%)	9 (30.0%)	37 (16.2%)	113 (20.8%)	
Received and needed	192 (24.0%)	5 (16.7%)	56 (24.4%)	131 (24.2%)	
Not received and needed	428 (53.4%)	13 (43.3%)	128 (55.9%)	287 (53.0%)	
**Had cut or reduced meals**	361 / 900 (40.1%)	18 / 35 (51.4%)	131 / 258 (50.8%)	212 / 607 (34.9%)	< 0.001
**Among those employed, ability to miss work**					
Was telecommuting or on paid leave	163 / 473 (34.5%)	8 / 23 (34.8%)	32 / 128 (25.0%)	123 / 322 (38.2%)	0.06
Cannot afford to miss work but was following confinement orders	146 (30.9%)	7 (30.4%)	40 (31.2%)	99 (30.8%)	
Cannot afford to stay home and must work to survive	164 (34.7%)	8 (34.8%)	56 (43.8%)	100 (31.1%)	

^a^ Denominators excluded individuals who did not respond or reported not knowing their answer unless otherwise noted

^b^ p-values were calculated using chi-squared and Fischer’s exact tests as appropriate

^c^ Denominator includes those who reported they "might or might not" lose their health insurance

A positive screen for depression was 1.63 (95% confidence interval [CI]: 1.36–1.97) times more common among those who had lost access to one or more gender-affirming resource during the COVID-19 pandemic as compared to those without reductions in access (**Table 5**). Similarly, a positive screen for anxiety was 1.61 (95% CI: 1.31–1.97) times more common among those who had lost access to one or more gender-affirming resource compared to those without reductions in access. Trends were similar for suicidal ideation. For example, among those with rare or no suicidal ideation at the beginning of the COVID-19 pandemic, individuals who had reduced access to gender-affirming resources were 1.74 (95% CI: 1.07, 2.82) times more likely to report increased suicidal ideation. Screening positive for depression and anxiety were also 1.21 (95% CI: 0.92, 1.58) and 1.48 (95% CI: 1.04, 2.10) times more prevalent among those reporting that the COVID-19 pandemic had decreased the amount of time they could live according to their gender, versus those who had increased that time. However, among individuals who had no or rare suicidal ideation prior to the COVID-19 pandemic, a smaller proportion of those living less according to their gender during the COVID-19 pandemic had increased suicidal ideation compared to those who lived more as their gender (prevalence ratio = 0.57 [95% CI: 0.33–0.98]).

**Table 5:** Bivariate prevalence ratios of screening positive for depression, screening positive for anxiety, and changes in suicidal ideation among transgender and nonbinary individuals from the COVID-19 disparities survey between April 16, 2020 and August 3, 2020.

	Screening positive for depression PrR (95% CI)^a^	Screening positive for anxiety PrR (95% CI)	Increased frequency of suicidal thoughts (vs. remained low)^b^	Retained frequent suicidal thoughts (vs. reduced frequency)^c^
Reported reduction in access to more than one (vs. 0) gender affirming resource	1.63 (1.36, 1.97)	1.61 (1.31, 1.97)	1.74 (1.07, 2.82)	1.37 (0.87, 2.15)
	n = 532	n = 523	n = 449	n = 112
Reported decreased time (vs. increased) lived according to one’s gender	1.21 (0.92, 1.58)	1.48 (1.04, 2.10)	0.57 (0.33, 0.98)	1.12 (0.50, 2.54)
	n = 346	n = 331	n = 310	n = 68

^a^ Prevalence ratio (PrR) and 95% confidence interval (95% CI)

^b^ Among individuals who reported never or seldom having suicidal ideation in the six months prior to the pandemic beginning

^c^ Among individuals who reported having suicidal ideation quite often, very often, and all the time in the sex months prior to the pandemic beginning

## Discussion

This survey provides insights into the early impacts of COVID-19 on gender-affirming resources, mental health, and economic stability among transgender and nonbinary communities across 76 countries. Roughly half of individuals reported that the emergence of SARS-CoV-2 had restricted their access to gender-affirming resources, and nearly two in five reported the COVID-19 crisis had negatively impacted their ability to live according to their gender. Screening positive for depression and/or anxiety and increases in suicidal ideation were common, but more so for those who experienced reduced access to gender-affirming resources. Of these resources, counseling and therapy were the most affected by COVID-19, but most people who responded to the survey also reported resiliencies, such as having sources of hope and being intent on finding emotional support. This intent to seek support, however, may be dampened by the fact that many individuals expected reductions in income and loss of health insurance. This is one of the first empirical studies to examine the effect of COVID-19 and its impact on gender-affirming resources, mental health, and economic stability among a sample of transgender and nonbinary individuals in low-, middle-, and upper-income countries across six continents.

Half of the individuals in this survey reported reduced access to one or more gender-affirming resource. This was similar to the TransCareCovid-19 online survey of 5,267 transgender and nonbinary individuals in upper and middle income countries, with most participants residing in Germany (23.9%) and the United Kingdom (10.3%) [29]. However, in our study, individuals from the European region reported more reductions in access to hormone therapy (40.5%) and surgical aftercare materials (39.8%) than reported in the TransCareCovid-19 survey (21% and 3% respectively). The TransCareCovid-19 survey also found over half of individuals had delayed or cancelled aftercare for a recent surgery. Differences may be due to the populations sampled, as our survey primarily drew from countries in Eastern Europe where there is substantial stigma against and policing of transgender individuals. Together, though, these results signify that the COVID-19 crisis is causing decreases in access to gender-affirming resources even in high-income settings.

Our study demonstrated that reduced access to gender-affirming resources due to the COVID-19 crisis were associated with poorer mental health. Screening positive for depression, screening positive for anxiety, and increased suicidal ideation were more common for those whose access to one or more gender affirming resource had been reduced due to the COVID-19 crisis. These results mirror anecdotal data from clinics serving transgender patients in China [41]. Relatedly, longitudinal data in the United States demonstrated that COVID-19-related disruptions to gender-affirming hormones and/or surgery were associated with increased psychological distress in a small cohort of transgender and nonbinary people [42].

The entire spectrum of gender-affirming resources and services— from haircuts to hormone therapy to surgery—are crucial to transgender and nonbinary individuals, as these resources and services activate and enhance the interactive process of receiving recognition for one’s gender, sense of self, and sense of humanity [43]. Given the abundant pre-COVID literature that gender affirmation also leads to better mental health and quality of life, our data underscore the importance of securing access to these essential resources and services to support the mental health of transgender and nonbinary individuals during the COVID-19 crisis [23, 24, 30, 44–46].

Access to gender-affirming resources should also be fortified to avoid negative physical health outcomes. For example, about a third of this sample reported reductions in access to hormones and 17% reported that they expected to lose their health insurance, which may support hormone therapy procurement. These disruptions may force some transgender and nonbinary individuals to discontinue hormone therapy or mete out their limited doses to last longer. Sustained interruptions or sub-optimal dosing of hormone therapy have been connected with symptoms of hypogonadism, such as osteoporosis and cardiovascular disease [47]. Similarly, aftercare for gender-affirming surgeries is critical for avoiding negative physical outcomes like urinary tract hesitancy and needing re-operation, yet a third of individuals in this study reported that they had reduced access to the surgical aftercare that they needed [48].

A third of individuals reported decreased time lived according to their gender. Living less according to one’s gender during the COVID-19 crisis was also associated with screening positive for depression and anxiety. People may be living in their genders less due to decreased social support, which has also been associated with psychological distress among transgender individuals during the COVID-19 crisis [42]. Among young (13–19 years), gender diverse people in the United States, 56% reported reduced ability to express their gender identity due to the COVID-19 crisis [49]. Youth often attributed needing to move back in with unsupportive caretakers as a reason for this change [31, 49].

However, a counterintuitive result was found among individuals in our study who had rare suicidal ideation prior to the COVID-19 crisis and lived more according to their gender during the COVID-19 crisis. This subset of the sample was more likely to have increased suicidal ideation as compared to individuals who were living according to their gender less. These results, however, may reflect a limitation of our measurement tool. For example, the former group likely had a low baseline ability to live according to their gender pre-pandemic and hence could only increase the amount of time lived according to their gender. The worse mental health in this group may be linked to low pre-pandemic levels of living according to their gender, rather than the recent increase in their ability to do so. Because we did not measure pre-pandemic and current ability to live according to one’s gender separately, we could not control for baseline levels in the model. It is also possible that people who recently began living in their gender more may be subjected to elevated levels of anticipated and experienced stigma that could be driving increased suicidality; prior research has shown discrimination due to gender expression, rather than gender identity itself, to be associated with mental distress [37, 50–52].

Positive screens for depression and anxiety were correlated with decreases in access to gender-affirming care and decreased time lived according to one’s gender, and were present in nearly half the sample. These data align with results from transgender and nonbinary youth from the Trevor Project poll and another sample of 201 young adults (19–25 years) attending college, both from the U.S. [49, 53]. Findings in a small study of 15 Latinx trans women in the United States suggest that these poor mental health indicators represent declines as a result of the COVID-19 crisis rather than just a high baseline prevalence [54]. These findings are particularly concerning when contrasted against the large proportion of individuals in this and other studies who reported that COVID-19 had decreased their access to mental health therapy and counseling [49, 53]. To mitigate the immediate trauma of the COVID-19 crisis and potential long-lasting effects, innovative mental health interventions— from remote video and phone therapy to self-help apps—have been emerging, but additional investments are needed, especially to reach those with limited to no access to Internet [55, 56]. Furthermore, our findings support the notion that transmasculine, transfeminine, and nonbinary populations are having distinct experiences during the time of COVID and should receive gender-specific support [41]. For example, transmasculine individuals were more likely to report having reduced access to gender-affirming resources but generally reported better mental health outcomes, as well as having sources of strength and comfort. Combined with the fact that transgender and nonbinary youth have been more likely to reach out to friends and family than cisgender lesbian, gay, and pansexual youth according to the Trevor Project data, it may be possible to enhance these resiliencies by strengthening and expanding trauma-informed, online peer-to-peer support efforts such as Q Chat Space [31, 49, 57]. However, different approaches to transfeminine, transmasculine, and nonbinary individuals may be needed.

Lastly, we found that the COVID-19 crisis is straining the core needs of transgender and nonbinary people worldwide, as it is for many other people— across finances, food, and employer-sponsored health insurance [58–60]. Such strain often follows inversely to pre-existing advantage or privilege within and across settings. For example, upwards of 77% of people from low-income countries in Africa and 37% of people in lower-middle-income countries reported work stoppage versus 26% in high-income countries [60, 61]. Such COVID-related inequities also exist within high-income countries, with access to healthcare and vaccines favoring upper-class families and other privileged groups [62]. For transgender and nonbinary communities, these strains intensify pre-existing economic vulnerabilities and will contribute to even greater barriers to gender-affirming care, mental health counseling, services, and products [27]. For example, approximately 10% of transgender and nonbinary individuals in the U.S. lacked health insurance before the COVID-19 pandemic [27]. Though some may have health insurance through their employers, transgender and nonbinary people are also more likely to be employed in the industries most impacted by business shutdowns [12]. We found that more than three quarters of the sample expected a reduction in their income, one in six expected to lose their health insurance, and more than half reported needing and not receiving financial aid. These results display the pronounced structural vulnerabilities that shape experiences of transgender and nonbinary communities in the current context of the COVID-19 pandemic, and likely contribute further as stressors to mental health. These stressors may climb as transgender and nonbinary communities continue to experience worsening economic instability due to the COVID-19 pandemic and associated control measures. Further research is necessary to examine the syndemic impact of COVID-19-related stressors on transgender and nonbinary communities, particularly those who are experiencing multiple, intersectional stressors at the individual, interpersonal, and structural levels.

This study included limitations. While the survey reached individuals from six continents and was available in 13 languages, these data are not a representative sample of transgender and nonbinary individuals worldwide. As a survey distributed through a mobile app, participation was limited to individuals with Internet and a smartphone. Individuals in the survey were also generally highly educated. Our survey likely missed highly disadvantaged individuals, and consequently, likely underestimates the true magnitude of impact of COVID-19 on this community. Our study also had a methodological limitation in its categorization of genders— while we aimed to offer an inclusive set of options, we did not capture the full spectrum nor fluidity of genders across the cultural diversity of the countries represented. Yet, our sample does present results from a large number of nonbinary individuals, which is a population that has generally been understudied but increasingly recognized as distinct from binary transgender individuals [63]. Furthermore, the survey only presented questions about access to gender-affirming resources to individuals who self-identified as transgender (e.g., versus those who selected being a man and were AFAB but did self-report as transgender). The next wave of data collection will present the module to all persons whose sex assigned at birth does not match their current gender.

The findings of the current study provide insights for future directions. Namely, future research should expand on this work by identifying protective factors that can be potentially leveraged to buffer the impact of COVID-19 pandemic on gender-affirming resources, mental health, and economic stability. There is also a need to contextualize and understand how transgender and nonbinary communities are currently responding to the economic instabilities due to the epidemic, particularly in regions where mandatory stay-at-home orders remain. These restrictions may have led or will lead some transgender and nonbinary individuals to turn to more dangerous work for income, unregulated and unmonitored markets for gender-affirming services or goods, or the use of alcohol and other substances to cope. Lastly, given the mobile and online nature of recruitment for this study, researchers should look for feasibility and opportunity with Hornet and other mobile apps as a platform for outreach, programming, and interventions for transgender and nonbinary communities in regions where the apps are utilized.

## Conclusion

Taken together, the finding reported here suggest the need for multiple programmatic interventions specific to transgender and nonbinary populations. Maintaining and increasing secure access to lifesaving gender-affirming resources, mental health services, and economic stability will require backing from both typical and atypical sources— from nonprofit organizations to for-profit companies to academic researchers— both during and after the COVID-19 pandemic. This could include, for example, instrumental support according to community needs (e.g., coordinating food bank deliveries or monetary support for bills) or resource mapping to help transgender and nonbinary individuals identify where they can seek pandemic-related relief and aid without stigma or discrimination [57]. Health insurers and healthcare facilities could also transparently communicate changes in policies and logistics for gender-affirming services to alleviate anxieties around loss of access due to pandemic control measures. To prevent detrimental mental health consequences due to inaccessibility of gender-affirming resources and economic hardships, rapid policies (e.g., digital prescription refills) and flexible interventions (e.g., telehealth) are needed to maintain continuity of gender-affirming hormones as well as therapy and counseling. To achieve these interventions, balanced partnerships will be needed to reach the most marginalized— both by supporting trans-led, community-based organizations to maintain and expand their transgender health services as well as by increasing the capabilities of nation- and/or state-sponsored programs and private sector companies to better serve transgender and nonbinary communities.

## Supporting information

S1 Table Access to and actualization of gender-affirming resources among self-identified transgender and nonbinary individuals who participated in the COVID-19 disparities survey, stratified by country (April 16 –August 3, 2020, N = 964). (DOCX)

S2 Table Mental health and resiliency indicators among transgender and nonbinary individuals who participated in the COVID-19 disparities survey, stratified by country (April 16 –August 3, 2020, N = 964). (DOCX)

S3 Table Economic indicators among transgender and nonbinary individuals who participated in the COVID-19 disparities survey, stratified by country (April 16 –August 3, 2020, N = 964). (DOCX)
