# Supplementary material for: Gender-affirming care, mental health, and economic stability in the time of COVID-19: A multi-national, cross-sectional study of transgender and nonbinary people
Source: PLoS One. 2021 Jul 9;16(7):e0254215. doi: 10.1371/journal.pone.0254215 (PMC8270151; doi:10.1371/journal.pone.0254215)
Supplement: S1 Table — (DOCX) [file pone.0254215.s001.docx]

**S1 Table. Access to and actualization of gender-affirming resources among self-identified transgender and nonbinary individuals who participated in the COVID-19 Disparities Survey, stratified by country (April 16 – August 3, 2020, N=964)**

|  | **European Region** | **South-East Asia Region** | **Region  of the Americas** | **Eastern Mediterranean Region** | **Western Pacific Region** | **African Region** | **p-value^a^** |
| --- | --- | --- | --- | --- | --- | --- | --- |
| **Experienced reduced access to  one or more gender affirming resource below^b^** | 116 / 207 (56.0%) | 109 / 204 (53.4%) | 32 / 53 (60.4%) | 30 / 56 (53.6%) | 6 / 25 (24.0%) | 15 / 23 (65.2%) | 0.043 |
| Hormone therapy and/or gender affirming medication | 47 / 116 (40.5%) | 55 / 186 (29.6%) | 10 / 25 (40.0%) | 11 / 32 (34.4%) | 4 / 19 (21.1%) | 8 / 13 (61.5%) | 0.081 |
| Surgical aftercare | 37 /93  (39.8%) | 51 / 175 (29.1%) | 7 / 15 (46.7%) | 8 / 33 (24.2%) | 2 / 18 (11.1%) | 8 / 12 (66.7%) | 0.008 |
| Cosmetic supplies and services, e.g., makeup, wigs, and hair removal | 68 / 176 (38.6%) | 62 /181 (34.3%) | 21 / 46 (45.7%) | 21 / 47 (44.7%) | 3 / 23 (13.0%) | 9 / 15 (60.0%) | 0.025 |
| Mental health counseling and therapy* | 62 / 128  (48.4%) | 73 / 186 (39.2%) | 25 /42 (59.5%) | 12 / 42 (28.6%) | 3 /19 (15.8%) | 9 / 18 (50.0%) | 0.004 |
| Body modifiers, e.g., binders and packing material | 56 / 134 (41.8%) | 58 / 179 (32.4%) | 17 / 40 (42.5%) | 14 / 46 (30.4%) | 3 / 21 (14.3%) | 8 / 13 (61.5%) | 0.030 |
| **Compared to before the COVID-19 pandemic, how often able to live according to their gender** |  |  |  |  |  |  |  |
| More or a lot more | 25 / 381  (6.6%) | 20 / 236  (8.5%) | 7 / 84  (8.3%) | 6 / 71  (8.5%) | 0 / 34  (0.0%) | 6 / 33  (18.2%) | 0.000 |
| About the same | 181  (47.5%) | 156  (66.1%) | 53  (63.1%) | 26  (36.6%) | 24  (70.6%) | 13  (39.4%) |  |
| Less or not at all | 175  (45.9%) | 60  (25.4%) | 24  (28.6%) | 39  (54.9%) | 10  (29.4%) | 14  (42.4%) |  |

^a^ p-values were calculated using chi-squared and Fischer's exact tests as appropriate

^b^ Denominators excluded participants who were not presented with these questions, did not respond, or said that the resource was not applicable to them
