# Supplementary material for: Gender-affirming care, mental health, and economic stability in the time of COVID-19: A multi-national, cross-sectional study of transgender and nonbinary people
Source: PLoS One. 2021 Jul 9;16(7):e0254215. doi: 10.1371/journal.pone.0254215 (PMC8270151; doi:10.1371/journal.pone.0254215)
Supplement: S2 Table — (DOCX) [file pone.0254215.s002.docx]

**S2 Table. Mental health and resiliency indicators among transgender and nonbinary individuals who participated in the COVID-19 Disparities Survey, stratified by country (April 16 – August 3, 2020, N=964)^a^**

|  | **European Region** | **South-East Asia Region** | **Region  of the Americas** | **Eastern Mediterranean Region** | **Western Pacific Region** | **African Region** | **p-value^b^** |
| --- | --- | --- | --- | --- | --- | --- | --- |
| **Screened positive per PHQ-4 (score ≥3)** |  |  |  |  |  |  |  |
| Depression | 232 / 400  (58.0%) | 66 / 222  (29.7%) | 46 / 82  (56.1%) | 48 / 76  (63.2%) | 21 / 40  (52.5%) | 18 / 32  (56.3%) | ≤ 0.001 |
| Anxiety | 392 / 856  (45.8%) | 392 / 856  (45.8%) | 392 / 856  (45.8%) | 392 / 856  (45.8%) | 392 / 856  (45.8%) | 392 / 856  (45.8%) | ≤ 0.001 |
| **Felt lonely since COVID-19 began** |  |  |  |  |  |  |  |
| Yes | 685 / 957  (71.6%) | 685 / 957  (71.6%) | 685 / 957  (71.6%) | 685 / 957  (71.6%) | 685 / 957  (71.6%) | 685 / 957  (71.6%) | 0.063 |
| **Frequency of suicidal ideation since  COVID-19 vs. 6 months prior** |  |  |  |  |  |  |  |
| Was and remains rare | 648 / 928  (69.8%) | 648 / 928  (69.8%) | 648 / 928  (69.8%) | 648 / 928  (69.8%) | 648 / 928  (69.8%) | 648 / 928  (69.8%) | 0.020 |
| Decreased from frequent to rare | 116 (12.5%) | 116 (12.5%) | 116 (12.5%) | 116 (12.5%) | 116 (12.5%) | 116 (12.5%) |  |
| Increased from rare to frequent | 93 (10.0%) | 93 (10.0%) | 93 (10.0%) | 93 (10.0%) | 93 (10.0%) | 93 (10.0%) |  |
| Was and remains frequent | 71 (7.6%) | 71 (7.6%) | 71 (7.6%) | 71 (7.6%) | 71 (7.6%) | 71 (7.6%) |  |
| **Reported having sources of hope, strength, comfort, and peace** |  |  |  |  |  |  |  |
| Yes | 489 / 768  (63.7%) | 489 / 768  (63.7%) | 489 / 768  (63.7%) | 489 / 768  (63.7%) | 489 / 768  (63.7%) | 489 / 768  (63.7%) | ≤ 0.001 |
| **Reported being intent on finding emotional support and therapy^c^** |  |  |  |  |  |  |  |
| Agree | 467 / 851  (54.9%) | 467 / 851  (54.9%) | 467 / 851  (54.9%) | 467 / 851  (54.9%) | 467 / 851  (54.9%) | 467 / 851  (54.9%) | ≤ 0.001 |
| Disagree | 136  (16.0%) | 136  (16.0%) | 136  (16.0%) | 136  (16.0%) | 136  (16.0%) | 136  (16.0%) |  |
| **Reported believing they could live a happy,**  **full life despite the pandemic^c^** |  |  |  |  |  |  |  |
| Agree | 516 / 847  (60.9%) | 516 / 847  (60.9%) | 516 / 847  (60.9%) | 516 / 847  (60.9%) | 516 / 847  (60.9%) | 516 / 847  (60.9%) | ≤ 0.001 |
| Disagree | 120  (14.2%) | 120  (14.2%) | 120  (14.2%) | 120  (14.2%) | 120  (14.2%) | 120  (14.2%) |  |

^a^ Denominators excluded individuals who did not respond or reported not knowing their answer unless otherwise noted
^b^ p-values were calculated using chi-squared and Fischer's exact tests as appropriate
^c^ Denominator includes those who stated "neither agree nor disagree"
