# Supplementary material for: Gender-affirming care, mental health, and economic stability in the time of COVID-19: A multi-national, cross-sectional study of transgender and nonbinary people
Source: PLoS One. 2021 Jul 9;16(7):e0254215. doi: 10.1371/journal.pone.0254215 (PMC8270151; doi:10.1371/journal.pone.0254215)
Supplement: S3 Table — (DOCX) [file pone.0254215.s003.docx]

**S3 Table: Socioeconomic indicators among transgender and nonbinary individuals who participated in the COVID-19 Disparities Survey, stratified by country (April 16 – August 3, 2020, N=964)^a^**

|  | **European Region** | **South-East Asia Region** | **Region of the Americas** | **Eastern Mediterranean Region** | **Western Pacific Region** | **African Region** | **p-value^b^** |
| --- | --- | --- | --- | --- | --- | --- | --- |
| **Lost job due to COVID-19** | 74 / 446  (16.6%) | 38 / 238  (16.0%) | 13 / 86  (15.1%) | 15 / 83  (18.1%) | 5 / 40  (12.5%) | 2 / 35  (5.7%) | 0.614 |
| **Expected reduction in income** |  |  |  |  |  |  |  |
| 0% | 113 / 436  (25.9%) | 39 / 340  (16.3%) | 25 / 86  (29.1%) | 19 / 79  (24.1%) | 9 / 40  (22.5%) | 9 / 35  (25.7%) | 0.055 |
| 1-39% | 123  (28.2%) | 66  (27.5%) | 20  (23.3%) | 20  (25.3%) | 14  (35.0%) | 11  (31.4%) |  |
| 40-99% | 140  (32.1%) | 110  (45.8%) | 34  (39.5%) | 25  (31.7%) | 13  (32.5%) | 10  (28.6%) |  |
| 100% | 60  (13.8%) | 25  (10.4%) | 7  (8.1%) | 15  (19.0%) | 4 (10.0%) | 5  (14.3%) |  |
| **Expected to lose health insurance^c^** |  |  |  |  |  |  |  |
| Yes | 32 / 307  (10.4%) | 42 / 165  (25.5%) | 11 / 67  (16.4%) | 16 / 51  (31.4%) | 7 / 35  (20.0%) | 1 / 21  (4.8%) | < 0.001 |
| No | 203  (66.1%) | 94  (57.0%) | 48  (71.6%) | 27  (52.9%) | 18  (51.4%) | 12  (57.1%) |  |
| **Receipt and need of financial aid** |  |  |  |  |  |  |  |
| Received and not needed | 10 / 375  (2.7%) | 6 / 208  (2.9%) | 2 / 75  (2.7%) | 3 / 64  (4.7%) | 0 / 32  (0.0%) | 0 / 30  (0.0%) | <0.001 |
| Not received but not needed | 86  (22.9%) | 29  (13.9%) | 19  (25.3%) | 13  (20.3%) | 6  (18.8%) | 5  (16.7%) |  |
| Received and needed | 53  (14.1%) | 92  (44.2%) | 23  (30.7%) | 8  (12.5%) | 8  (25.0%) | 5  (16.7%) |  |
| Not received and needed | 226  (60.3%) | 81  (38.9%) | 31  (41.3%) | 40  (62.5%) | 18  (56.3%) | 20  (66.7%) |  |
| **Had cut or reduced meals** | 167 / 420  (39.8%) | 82 / 230  (35.7%) | 28 / 82  (34.2%) | 45 / 75  (60.0%) | 12 / 37  (32.4%) | 16 / 34  (47.1%) | 0.004 |
| **Among those employed,  ability to miss work** |  |  |  |  |  |  |  |
| Was telecommuting or on paid leave | 81 / 214  (37.9%) | 40 / 127  (31.5%) | 17 / 42  (40.5%) | 11 / 34  (32.4%) | 6 / 21  (28.6%) | 6 / 20  (30.0%) | 0.165 |
| Cannot afford to miss work but was  following confinement orders | 72  (33.6%) | 34  (26.8%) | 9  (21.4%) | 9  (26.5%) | 5  (23.8%) | 10  (50.0%) |  |
| Cannot afford to stay home and must  work to survive | 61  (28.5%) | 53  (41.7%) | 16  (38.1%) | 14  (41.2%) | 10  (47.6%) | 4  (20.0%) |  |

^a^ Denominators excluded individuals who did not respond or reported not knowing their answer unless otherwise noted
^b^ p-values were calculated using chi-squared and Fischer's exact tests as appropriate
^c^ Denominator includes those who reported they "might or might not" lose their health insurance
